# Glymphatic system function in patients with ischemic stroke evaluated by the DTI-ALPS method: a comprehensive review

**DOI:** 10.3389/fneur.2025.1607723

**Published:** 2025-09-23

**Authors:** Lichuan Zeng, Zihan Yin, Wei Li, Xiao Wang, Yaodan Zhang, Mingguo Xie, Ling Zhao

**Affiliations:** ^1^Acupuncture and Tuina School, Chengdu University of Traditional Chinese Medicine, Chengdu, China; ^2^Department of Radiology, Hospital of Chengdu University of Traditional Chinese Medicine, Chengdu, China; ^3^Department of Radiology, Deyang Hospital Affiliated Hospital of Chengdu University of Traditional Chinese Medicine, Deyang, China; ^4^Department of Neurology, Hospital of Chengdu University of Traditional Chinese Medicine, Chengdu, China

**Keywords:** glymphatic system, ischemic stroke, diffusion tensor imaging-analysis along perivascular space, perivascular space, MRI

## Abstract

The glymphatic system is a glial-dependent waste clearance pathway in the central nervous system (CNS) of vertebrates that exploits the perivascular compartment between the vascular basement membrane (outermost wall of blood vessels) and astrocytic vascular endfeet to facilitate exchange between cerebrospinal fluid and interstitial solutes throughout the brain. This intricate network plays a vital role in the efficient elimination of metabolic waste and the regulation of water transport within the brain. Ischemic stroke, characterized by interrupted or reduced blood supply to a specific region of the brain, is a major cause of disability and mortality. Impairment of the glymphatic system is implicated in the pathophysiological process of stroke, including disruption of the blood–brain barrier, formation of cerebral edema, induction of neuroinflammation, and accumulation of neurotoxic factors. Various studies have demonstrated asymmetry and impairment of glymphatic function during ischemic stroke. Diffusion tensor image analysis along the perivascular space (DTI-ALPS) using diffusion magnetic resonance imaging is an effective method for evaluating glymphatic system function by examining interstitial fluid dynamics within the human brain. In this study, we provide an overview of putative mechanisms mediating the role of the glymphatic system in ischemic stroke pathophysiology, with a strong focus on discussing DTI-ALPS applications in assessing changes in glymphatic function following an ischemic stroke.

## 1 Introduction

Stroke, clinically classified into ischemic or hemorrhagic subtypes, is one of the most common causes of mortality and disability globally, with rising incidence in developing countries (1). Acute ischemic stroke (AIS) is responsible for about 71% of all strokes globally (2) and is predominantly caused by cerebrovascular obstruction (3, 4). Uncontrolled hypertension, cardiac diseases, and large artery atherosclerosis have been established as the primary etiologies of ischemic stroke (5–7), with small vessel disease being another significant contributor to stroke (8). In recent years, there has been a growing focus on elucidating the physiological roles of the glymphatic system (9–12). The intricate network of the glymphatic system facilitates cerebrospinal fluid (CSF) transport via perivascular channels within the brain and is believed to play a crucial role in metabolic waste clearance. In addition to its role in waste clearance, the glymphatic system may also facilitate the distribution of essential substances, such as glucose, lipids, amino acids, and neurotransmitters, throughout the brain. The discovery of the glymphatic system produced a shift in perspective regarding the pathology of neurodegenerative diseases and acute neurological disorders such as stroke (13, 14). Studies of the glymphatic system have largely been conducted using tracer methodologies, with gadolinium-based contrast agent (GBCA) administration via the intrathecal route considered the gold standard for tracer investigations using magnetic resonance imaging (MRI) in human studies. However, the GBCA-enhanced MRI technique is invasive and lacks regulatory approval. In contrast, diffusion tensor image analysis along the perivascular space (DTI-ALPS) offers a noninvasive approach to investigating the human glymphatic system and has recently been applied in Alzheimer’s disease, cerebral small vessel disease, and sleep-related diseases (15–18). The DTI-ALPS has been proposed as an index for quantifying water diffusivity patterns along the deep medullary vein at the level of the lateral ventricular body. In this review, we discuss the physiology of the glymphatic system and the related mechanisms involved in post ischemic injury, which could provide a new direction for research on ischemic stroke. Furthermore, we discuss DTI-ALPS as an index for assessing glymphatic system function in patients who have experienced ischemic stroke.

## 2 Current understanding of the glymphatic system

### 2.1 Glymphatic drainage

The current understanding of intracerebral lymphatic drainage systems encompasses three components: the glymphatic system, the intramural periarterial drainage pathway (including the influx and efflux pathways), and the meningeal lymphatic vessels (9, 10). The glymphatic system was initially described as a macroscopic waste clearance mechanism that employs astroglial (glial-lymphatic) channels to eliminate soluble proteins and metabolites from the CNS, and, in fact, it facilitates the exchange of fluid and solutes between the CSF and the interstitial compartments of the brain. Nonetheless, its role in brain function extends beyond waste clearance to encompass lactic acid, tau protein, amyloid-β and α-Synuclein. Furthermore, the glymphatic system plays a pivotal role in the exchange of glucose, nutrients, neurotransmitters, and neuroactive substances between the CSF and interstitial compartments of the brain (19, 20).

**Figure 1:**
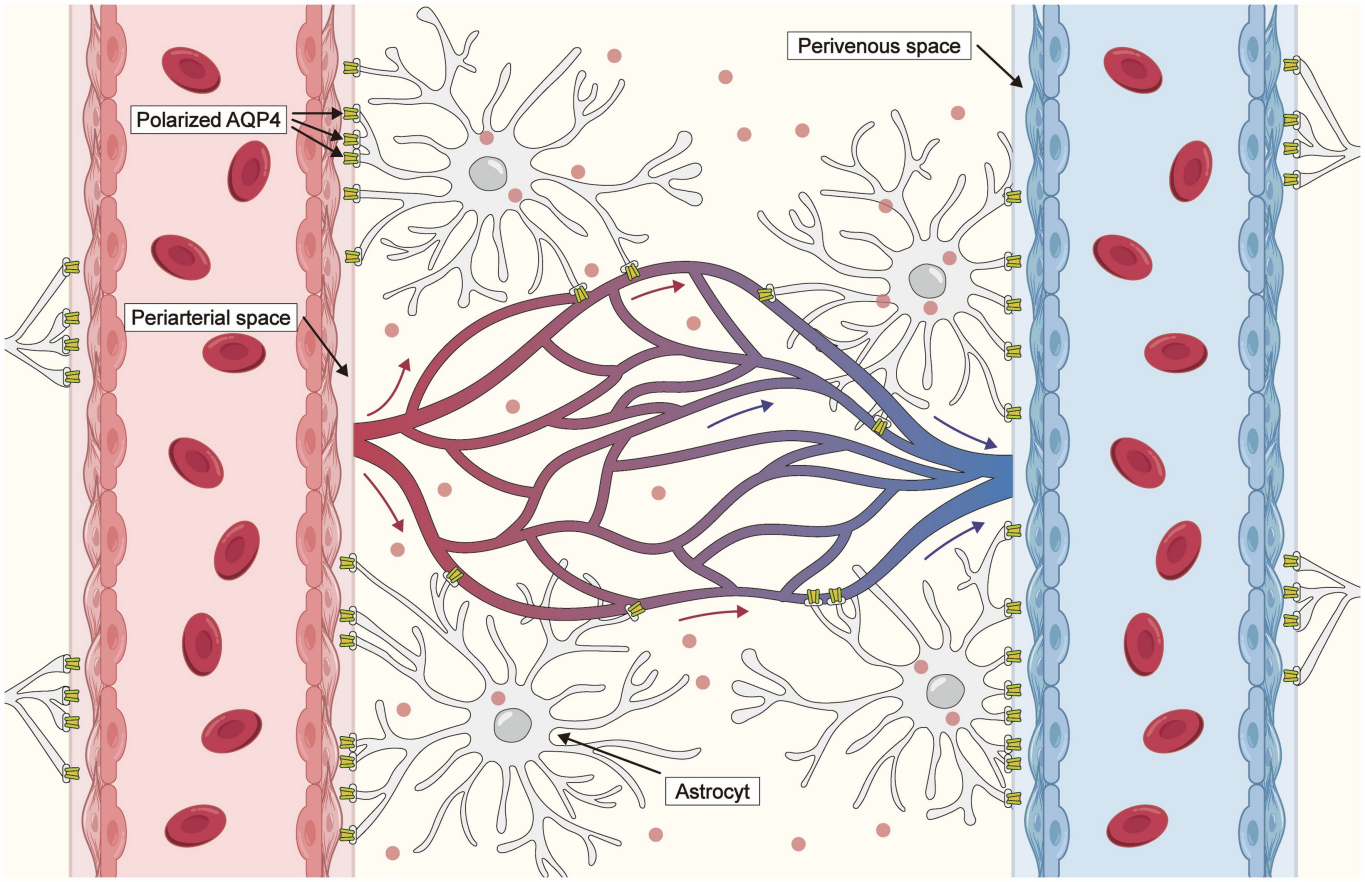
Anatomical structure and functions of the glymphatic system. Cerebrospinal fluid (CSF) flows from the subarachnoid space into the cerebral parenchyma through the periarterial space. Subsequently, CSF is exchanged with interstitial fluid (ISF), facilitated by *polarized* aquaporin-4 (AQP4) water channel expression on the adjacent astrocytic end feet, driving a convective flow of interstitial solutes and ISF into perivenous spaces to effectively remove metabolic waste products from the.

The glymphatic system was first reported by Iliff et al. (21) in a study in which the pathway of an intracisternally injected fluorescent tracer was detected using two-photon laser scanning microscopy, enabling real-time visualization of the CSF influx route in anesthetized mice. After injection, the tracers swiftly traversed the perivascular space surrounding the penetrating arterioles and cortical surface arteries before permeating into the brain parenchyma, thereby providing visual representation of the interchange of CSF and interstitial fluid (ISF). The glymphatic system comprises three crucial compartments: a periarterial route for CSF influx, a perivenous pathway for ISF efflux, and an astrocytic aquaporin-4 (AQP4)-dependent exchange pathway within the parenchyma (Figure 1). The perivascular spaces (PVSs)—including the periarteriolar, pericapillary, and perivenular spaces—are small CSF-filled regions between blood vessels and the pia mater that facilitate ISF drainage. Brain PVSs are part of the glymphatic system and facilitate the clearance of metabolic byproducts. Recent research findings indicate that there is a continuous exchange of CSF and ISF facilitated by convective influx along the periarterial space (22–24). CSF flows from the subarachnoid space into the brain parenchyma via the periarterial spaces of the penetrating arteries, facilitated by aquaporin-4 water channels (25). Subsequently, the CSF intermixes with parenchymal ISF. The solutes present in the ISF and their accompanying movement then transit into the perivenous and perineuronal spaces before exiting the brain parenchyma. Once the CSF-ISF mixture reaches the subarachnoid space, it traverses the arachnoid granulations and drains into the dural sinuses, meningeal lymphatics, and cervical lymphatics (26, 27).

The existence of lymphatic vessels and drainage pathways has been confirmed through imaging and histological experiments on humans and animals. Absinta et al. (28) identified lymphatic vessels in the human dura mater. In their study, they administered intravenous injections of gadobutrol and found gadolinium accumulating in meningeal lymphatic vessels (mLVs) in healthy volunteers. These vessels absorb ISF and serve as a pathway for transporting it to deep cervical lymph nodes. Aspelund et al. (29) discovered a dural lymphatic network that drains CSF from the subarachnoid space, as well as brain ISF via the glymphatic system, conveying these fluids to the deep cervical lymph nodes. Through research involving the anatomical dissection of human cadavers, Pessa (30) identified a new structure in the brain’s sagittal sinus. This structure, called the CSF canalicular system, comprises channels on either side of the sagittal sinus vein that connect to the subarachnoid CSF via the Virchow-Robin spaces. These findings align with earlier reports describing CSF channels in the neck that extend from the cranial base to the subclavian vein. The most recent study in this domain also confirms that CSF flows into the initial lymphatics in the meninges at the base of the skull and then subsequently flows through the extracranial periorbital, olfactory, nasopharyngeal, and hard palate lymphatics, proceeding via smooth muscle-covered superficial cervical lymphatics to the submandibular lymph nodes (31).

### 2.2 AQP4 in the glymphatic system

In the glymphatic system, astrocytic vascular endfeet enveloping the cerebral vasculature are interconnected through gap junctions. Bork et al. (32) propose that astrocyte endfeet may also serve as valves for converting pressure oscillations into directed fluid flow. Aquaporins (AQPs), integral membrane proteins that facilitate the selective transport of water and solutes across cell membranes, have been the subject of extensive research. AQPs play a crucial role in maintaining cellular homeostasis and fluid balance within neural compartments, and have attracted substantial research attention given the potential implications of such a role in both physiological and pathological contexts (33). AQP4 is primarily expressed in the plasma membrane of astrocytes; however, it exhibits distinct localization to specialized regions such as astrocyte endfoot processes. These polarized expression patterns are postulated to be regulated by the intracellular interrelationship between AQP4 and α-syntrophin. Glymphatic system activity is highly dependent on the polarized localization of AQP4. Disruption of AQP4 polarization has been implicated in various degenerative and acute brain pathologies.

A portion of CSF originating from the subarachnoid space enters the parenchyma via the PVS surrounding arteries, permeating into the brain parenchyma alongside arterioles, capillaries, and venules. CSF within the PVS of arterioles and capillaries intermixes with the ISF, facilitating the drainage of metabolic waste from the brain via perivenous spaces. This process is facilitated by the polarized expression of AQP4 on the astrocyte endfeet facing the perivascular space. This AQP4-mediated glymphatic drainage process is important in the formation and resolution of cerebral edema following brain injury (34). A previous study reports that the genetic deletion of AQP4 leads to a roughly 65% impairment in CSF-ISF exchange and a 55% reduction in the clearance of β-amyloid (35). These findings indicate that the AQP4-mediated glymphatic pathway plays a critical role in eliminating ISF solutes—including waste products—from the brain parenchyma.

### 2.3 Factors influencing the glymphatic system

Multiple factors have the potential to influence CSF drainage through the glymphatic system. Cerebral arterial pulsation is the primary driving force for CSF influx in the glymphatic system (36–38) and was initially observed following unilateral internal carotid artery ligation that resulted in a reduction in cerebral arterial pulsation and decelerated perivascular CSF-ISF exchange. Administering dobutamine increases the cerebral arterial pulsation rate and facilitates perivascular CSF-ISF exchange (36). Plog et al. (39) demonstrated that penetrating arterial pulsatility decreases significantly and bilaterally after unilateral craniectomy in mice, leading to both immediate and long-term impairment of glymphatic CSF influx in both the ipsilateral and contralateral brain parenchyma. Arterial pulsation is an important pump for CSF influx into the parenchyma and CSF-ISF exchange. Pulsations in the large arteries continuously propagate pressure waves along the major vessels. As an artery extends into the CSF-filled subarachnoid space, a portion of the ejection pressure is transformed into kinetic energy, which drives CSF convection, thereby facilitating CSF movement into the parenchyma via periarterial spaces.

Furthermore, the respiratory-related pulsatile cycle promotes centripetal venous fluid flow, augmenting perivenous spaces and driving CSF outflow through the glymphatic system (40). In addition, the venous–arterial pressure gradient, enhanced by respiratory cycles, drives the CSF-ISF exchange (41, 42). Subsequent studies have found that glymphatic activity exhibits a diurnal rhythm, with the clearance of toxic compounds mediated by the glymphatic system occurring primarily during sleep. Using two-photon imaging, Xie et al. (43) discovered a 90% increase in glymphatic function during sleep in live mice. In contrast, glymphatic function was significantly suppressed during wakefulness. During sleep, the interstitial space expanded by 60%, resulting in a remarkable enhancement of the CSF-ISF exchange and amyloid-beta (Aβ) clearance. Age is another factor that influences the glymphatic system. The clearance of intraparenchymally injected amyloid-beta has been found to be impaired by 40% in aged mice relative to young ones. This decline in CSF-ISF exchange in mice is accompanied by a significant reduction of 27% in intracortical arteriole wall pulsatility and widespread loss of perivascular AQP4 polarization along the penetrating arteries (44). It has been surmised that glymphatic flow diminishes with age, resulting in the accumulation of metabolic waste. This accumulation leads to occlusions along the glymphatic pathway and further exacerbates impairment of the glymphatic flow (23, 24, 45).

## 3 Glymphatic dysfunction in ischemic stroke

**Figure 2:**
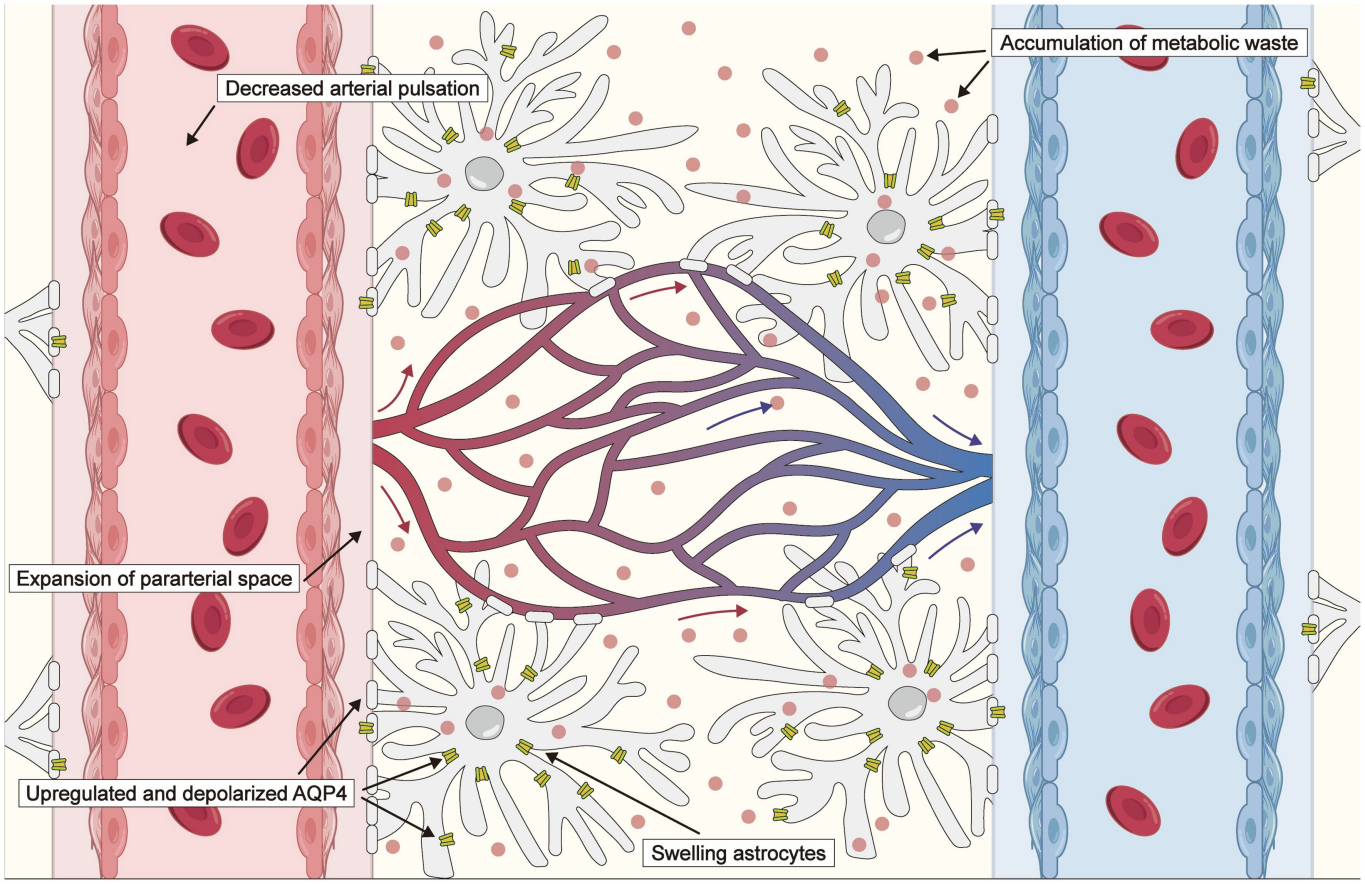
Schematic of changes in the glymphatic system after an ischemic stroke. The driving force is reduced due to decreased arterial pulsation following an ischemic stroke. Expansion of the para-arterial spaces facilitates an influx of cerebrospinal fluid (CSF) toward the interstitium, which is associated with exacerbated cytotoxic edema. Aquaporin 4 (AQP4) distribution transfers from the perivascular endfeet to the entire astrocytic membrane, and AQP4 expression is upregulated, impairing the clearance of CSF, metabolites, and inflammatory cytokines.

Hemodynamic dysfunction, characterized by reduced cerebral blood flow, impaired cerebrovascular reactivity, and altered vascular pulsatility, underlies the progression of ischemic stroke. Considerable attention has been devoted to investigating the relationship between lymphatic drainage systems and ischemic stroke, particularly the involvement of lymphatic drainage systems in the development of cerebral edema, modulation mechanisms underpinning changes in AQP4, accumulation of toxic factors, and the activation of neuroinflammation after stroke (46–48). Following an infarction, neurons undergo depolarization events, resulting in the loss of their transmembrane potentials (49), which leads to significant vasoconstriction in the smooth muscle cells of both parenchymal and leptomeningeal arteries due to the release of K^+^ and vasoactive substances (50). Consequently, this process expands the para-arterial spaces, facilitating an influx of CSF toward the interstitium. When the influx of CSF into the cerebral parenchyma—facilitated by the glymphatic system—is augmented and the efflux of ISF is impeded, aberrant accumulation of cerebral tissue fluid may ensue, leading to brain edema. A schematic of the changes in the glymphatic system after an ischemic stroke is provided in Figure 2.

### 3.1 Cerebral edema

Damage to the blood–brain barrier (BBB) has traditionally been held to influence the occurrence and development of poststroke brain edema. The BBB is compromised following an ischemic stroke, leading to extravasation and accumulation of water and macromolecular substances within perivascular and interstitial cells (14, 51). Cerebral edema is a pathological phenomenon characterized by an increase in brain water content and volume, and it is a prevalent characteristic observed in various brain diseases—including stroke, inflammation, brain tumors, and traumatic brain injuries. Brain edema following ischemic stroke can generally be classified into two distinct stages: an initial stage characterized by cytotoxic and ionic edema, and a subsequent stage marked by vasogenic edema, which is associated with an impaired BBB.

The dysfunction occurring 3 h after ischemic stroke in mice models has been attributed to attenuated vascular pulsation and occlusive perivascular space caused by thrombus formation—however, arterial recanalization after 24 h can restore glymphatic system function (52). Notably, glymphatic dysfunction may result in futile recanalization, a phenomenon in which recanalization fails to restore glymphatic system function in up to 50% of patients. Several studies are currently investigating the modulation of AQP4 function following an infarction to reduce cerebral edema and decrease the likelihood of futile recanalization, thereby improving functional outcomes (53, 54). Elucidating the molecular mechanisms and the role of the glymphatic system in the pathophysiology of ischemic stroke may facilitate the development of therapeutic interventions that enhance poststroke functional recovery. Zhu et al. (55) have demonstrated a severe impairment of paravascular CSF influx and glymphatic function following middle cerebral artery occlusion in mice. To investigate the causal relationship between glymphatic system function and brain edema further, they employed adrenergic inhibition to enhance glymphatic system function, resulting in significant reductions in hemispheric edema volume and Aβ deposition. Conversely, when they suppressed glymphatic system function using an adrenergic agonist, brain edema was significantly augmented on Day 7. Their findings indicate that the progression and resolution of brain edema coincide with the impairment and recovery of glymphatic system function, highlighting the crucial role of glymphatic system transport in brain edema induced by ischemic stroke.

Another animal study found that glymphatic function is reduced during the acute phase following an ischemic stroke—specifically, between the first 24 h and Day 7—with subsequent recovery observed after that phase (48). A recent study demonstrated that diffuse ischemia induces CSF influx into the perivascular space within minutes following AIS in mice, with this being the primary mechanism underpinning immediate edema (56). This CSF influx drives acute tissue swelling, a pathogenic process initiated by progressive depolarization and subsequent vasoconstriction, which in turn enlarges the perivascular spaces and doubles the glymphatic inflow speeds. Furthermore, enlargement of the PVS has been linked to the pathogenesis of numerous other neurological disorders (57). It has been postulated that rapid depolarization caused by brain ischemia leads to enlargement of the PVS and increased glymphatic flow, which might be one of the pathological mechanisms that underpin tissue swelling and cerebral edema (58).

### 3.2 AQP4 abnormality in ischemic stroke

AQP4 expression is predominantly localized in the astrocyte endfeet surrounding blood vessels, with less frequent localization observed in cell bodies and polarized distribution. The polarized distribution of AQP4 ensures optimal functioning of the glymphatic system. However, AQP4 may exert pro-inflammatory effects by mediating AQP4-dependent astrocyte swelling and cytokine release. Several studies have demonstrated that the absence of AQP4 confers protection to the CNS and mitigates neuroinflammation (59, 60). The beneficial effect of reduced AQP4 expression stems from the resulting decrease in cytotoxic brain water accumulation and the attenuation of endfeet swelling in astrocytes. However, abnormal AQP4 expression may occur after an ischemic stroke.

Kitchen et al. (61) found that AQP4 levels were upregulated in response to hypoxia-induced cell swelling through a calmodulin-dependent mechanism in rats. Calmodulin directly interacts with the carboxyl terminus of AQP4, inducing a specific conformational change and promoting its localization on the cell surface. In addition, protein kinase A can facilitate nuclear translocation of the transcription factor Foxo3a, which directly activates the expression of the AQP4 gene and subsequently enhances AQP4 expression. Ribeiro et al. (62) demonstrated the presence of two distinct peaks in hemispheric swelling occurring at 1 h and 48 h post-ischemia in mice, which coincided with the observed peaks in AQP4 expression. Specifically, a significant increase in AQP4 expression was observed 1 h after occlusion, localized primarily to the astrocyte endfeet within both the core and border regions of the lesion. Furthermore, an additional increase in AQP4 expression was noted at 48 h in astrocytes located at the border region of the lesion. Their study findings suggest that AQP4 might serve as the main route of fluid movement after cerebral ischemia.

After an ischemic stroke, the pattern of AQP4 distribution shifts from predominance in the perivascular endfeet to the entire astrocytic membrane, and AQP4 expression increases, potentially promoting astrocyte migration and glial scar formation—which is not conducive to perivascular polarization of AQP4 (63, 64). Zhu et al. (55) found that AQP4 polarization around the microvascular structures was significantly decreased on Day 2 after middle cerebral artery occlusion across infarcted core and peri-infarct regions. Sun et al. (65) demonstrate that acute inhibition of AQP4 using N-(1,3,4- thiadiazol-2-yl) pyridine-3-carboxamide dihydrochloride (TGN-020) in rats facilitates neurological recovery by reducing cerebral edema during the early stage, and mitigating peri-infarct astrogliosis and AQP4 depolarization during the subacute phase following stroke. These studies also reveal that the extent of progressive edema is dependent on the expression of AQP4 channels.

AQP4 abnormalities may alter the water permeability across the cell membrane. Noninvasive diffusion-weighted imaging (DWI) has been utilized extensively as a key tool for assessing AQP4 abnormalities. The apparent diffusion coefficient (ADC) reflects the overall diffusion signal resulting from multiple diffusion-related factors. The diffusion signal may be influenced by changes in AQP4 expression, changes in intracellular and extracellular water content, the extent of demyelination, and the severity of cerebral edema, depending on the specific diffusion characteristics of the tissue. Urushihata et al. (66) compared ADCs estimated for ischemic and normal tissue in AQP4 knockout and wild-type mice. For the ischemic region, AQP4 knockout mice exhibited lower ADC values than wild-type mice, while higher ADC values were observed in the contralateral region. The observation of reduced ADC values in AQP4 knockout mice aligns with a previous study that reported an increase in ADC following the administration of the aquaporin-4 inhibitor TGN-020 (67). Furthermore, low *b*-value DWI has been employed to evaluate the characteristic CSF dynamics. In a study by Taoka et al. (68) involving patients with ventricular dilatation, CSF signal intensity in DWI at b = 500 s/mm^2^ was examined in the lateral, third, and fourth ventricles, as well as in the cerebral sulci and the Sylvian fissure. The measured values revealed a significantly low CSF signal intensity—specifically in the lateral and third ventricles of these patients—indicating altered CSF dynamics in these regions.

### 3.3 Neuroinflammation and the accumulation of metabolic waste

After an ischemic infarct, ISF clearance in the glymphatic system is reduced (69, 70) Meanwhile, necrotic cells release damage-associated molecular patterns into the extracellular environment, triggering the activation of microglial cells. Subsequently, activated microglia secrete a considerable number of pro-inflammatory mediators that potentiate neuroinflammation. Zbesko et al. (71) found that extracellular fluid in areas of liquefactive necrosis following stroke exhibits long-lasting toxicity to primary cortical and hippocampal neurons that persists for at least 7 weeks poststroke. Furthermore, they confirmed the permeability of these toxic molecules across the glial scar and their subsequent removal through a combination of paravascular clearance and microglial endocytosis in adjacent tissues. These findings suggest that increased glial scar permeability is one of several mechanisms underpinning neurodegeneration following a stroke.

Sun et al. (72) applied near infrared-II (NIR-II) nanoprobes in mice research to investigate impaired glymphatic influx and reduced glymphatic efflux, and confirmed compromised glymphatic function following cerebral ischemia. The high-penetration NIR-II fluorescence signal enables rapid assessment of the size of endfoot tubes and the influx of the NIR-II tracer into the brain. This represents a promising approach for investigating the glymphatic system in rodent models and evaluating its potential as a target for stroke diagnosis and treatment. The glymphatic system plays a crucial role in flushing toxic products out of the CNS after ischemic events, and it has been hypothesized that post-ischemic stroke, inflammatory processes involving the PVS and BBB, and impaired waste clearance affecting the glymphatic system may occur post-ischemia. Reactive astrogliosis and glial scar formation are the principal pathological characteristics observed in the brain following ischemic stroke. Reactive astrogliosis typically manifests within 48–96 h following ischemia and is characterized by the upregulation of glial fibrillary acidic protein (GFAP) expression, cellular hypertrophy, and the subsequent development of a glial scar that impedes neuronal regeneration (73).

Impairment of the glymphatic system may lead to the accumulation of toxic solutes and proteins within the infarcted area. It has been proposed that the accumulation of neutrophils in the ischemic core, along with the activation and proliferation of microglia in the penumbra, occurs within the first 72 h (74). Furthermore, numerous studies have focused on the immune response to acute cerebral ischemia as a pivotal determinant in the pathogenesis of brain lesions and neurological deficits. This immune response primarily encompasses the activation of resident glial cells accompanied by the infiltration of circulating leukocytes. In addition to the compromised BBB, meningeal lymphatics serve as an alternative route for peripheral immune cell infiltration into the brain parenchyma, exacerbating neuroinflammation during ischemic stroke. Therefore, it is postulated that meningeal lymphatics play a pivotal role in determining the outcome of ischemic stroke (75, 76).

## 4 Principles of the DTI-ALPS method

Diffusion denotes the broad movement of matter, wherein molecules or ions intermingle through natural agitation in an unpredictable manner. DWI offers an image contrast hinged on the molecular dynamics of water and is characterized by heightened sensitivity and precision in identifying acute ischemia (67, 77). Cerebral ischemia leads to a reduction in the diffusion of water molecules within the affected area, accompanied by a swift decrease in ADC values, and is attributable to a coalescence of intricate biophysical factors. Three primary mechanisms have been delineated: (1) alterations in the relative volumes of intracellular and extracellular compartments, (2) augmented tortuosity of the extracellular space, and (3) reduced cytoplasmic flow/microstreaming within the intracellular milieu (77). The mechanism underlying the changes in diffusion during cerebral ischemia involves the disruption of energy metabolism, which leads to the failure of the Na^+^/K^+^ ATPase pump (78). This dysfunction results in the net movement of water from the extracellular space into the intracellular environment, a phenomenon known as cytotoxic edema. In this state, the movement of water is considerably restricted, correlating with diminished ADC values. As the infarct progresses into the acute and subacute stages, vasogenic edema develops, causing an increase in tissue water, primarily within the extracellular compartment, while the intracellular water remains relatively stable due to persistent cytotoxic edema. Consequently, there is a gradual rise in ADC values attributable to cellular lysis and escalating vasogenic edema.

MRI with diffusion sequences has emerged as a noninvasive diagnostic tool for evaluating the human glymphatic system. The use of intrathecal administration of GBCA as a tracer to study cerebral ISF dynamics and waste elimination is currently limited. The DTI-ALPS method was first described by Taoka et al. (79) in a study evaluating glymphatic system activity in patients with Alzheimer’s disease. The motion of water molecules within the perivascular space was assessed by measuring diffusivity using diffusion tensor imaging (DTI). The medullary arteries and veins are cerebral vessels located within the parenchyma that run adjacent to the perivascular space, which serve as the main conduit for fluid drainage in the glymphatic system. DTI-ALPS enables noninvasive investigation of the glymphatic system, as well as Parkinson’s disease, small vessel diseases, and traumatic brain injury—with a low DTI-ALPS index value indicating reduced activity (80–82). The glymphatic system is influenced by various factors, including arterial pulsation, blood pressure, venous sinus pressure, cerebral edema, and AQP4 expression. DTI-ALPS may capture CSF flow and, potentially, a composite of the contributing factors. There are three paradigms for calculating the DTI-ALPS index: (1) using a DTI-unguided atlas-based approach, (2) using DTI-guided manually delineated regions of interest (ROIs) in fiber tracts, and (3) using spherical ROIs placed within the projection and association fibers. The latter two methods provide greater statistical power than the first method. The DTI-unguided approach often includes water diffusivity measurements from regions beyond the perivascular spaces (PVS), which may dilute the DTI-ALPS index (83).

**Figure 3:**
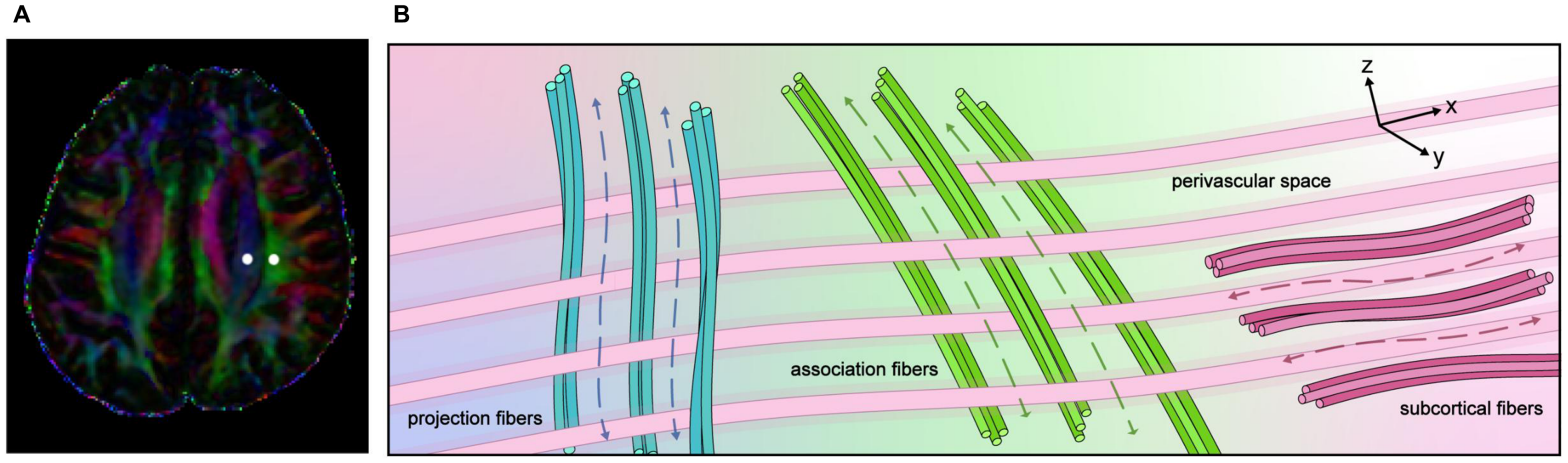
The concept of the diffusion tensor image analysis along with the perivascular space (DTI-ALPS) method. **(A)** Superimposed color display of DTI, indicating the distribution of projection fibers (blue area), association fibers (green area), and the subcortical (red area), and an example of region of interest (ROI) placement. **(B)** Schematic diagram illustrating the spatial relationship between the orientation of the perivascular space and the directions of the projection and association fibers. It is evident that the perivascular space is oriented perpendicular to both the projection and association fibers.

At the level of the lateral ventricular body, the medullary vessels and their PVS run in the right–left direction (*x*-direction), which is also perpendicular to the direction of both the projection fibers (mostly oriented in the *z*-direction) and the association fibers (mostly oriented in the *y*-direction). The basic concept of the along the perivascular space (ALPS) method is the evaluation of water movement in this x-direction. The diffusion of large nerve fibers dominates in diffusion images, and the diffusivity along the *x*-direction in regions with projection/association fibers will at least partly reflect the diffusivity along the perivascular space. This method calculates the ratio of diffusion in the direction of the PVS in relation to the diffusion of free water in the interstitium. The concept of the DTI-ALPS method is presented in Figure 3.

The DTI-ALPS index is provided by the ratio of two sets of diffusivity values, which are perpendicular to the dominant fibers in the tissue—that is, the ratio of the mean of D*_xxproj_* and D*_xxassoc_* to the mean of D*_yyproj_* and D*_zzassoc_.* The DTI-ALPS index can be expressed as follows:


$$\mathrm{DTI}-\text{ALPS index}=\frac{\text{mean}\;(D_{\text{xxproj}},D_{\text{xxassoc}})}{\text{mean}\;(D_{\text{yyproj}},D_{\text{zzassoc}})}$$

where D*_xxproj_* is the x-axis diffusivity in the area of the projection fibers, Dxxassoc is the x-axis diffusivity in the area of the association fibers, D*_yyproj_* is the y-axis diffusivity in the area of the projection fibers, and D*_zzassoc_* is the z-axis diffusivity in the area of the association fibers (84). An elevated DTI-ALPS index indicates the predominance of water diffusion in the x-direction; conversely, a low DTI-ALPS index indicates the absence of dominance of water movement in the x-direction. The reproducibility of a DTI-ALPS study demonstrated that the DTI-ALPS index is robust under a fixed imaging method, even when different scanners are used (85). However, the replication study reported a statistically significant difference in the DTI-ALPS index values obtained with evaluations using three different echo times (TEs). Furthermore, there were statistically significant differences in the DTI-ALPS index values for different numbers of MPG axes (i.e., 12 axes vs. 30 axes).

## 5 DTI-ALPS for ischemic stroke

A study by Zhang et al. (86) on the DTI-ALPS index and intrathecal contrast administration evaluated glymphatic system function and reported a strong correlation between the two methods. The noninvasive and rather simple nature of this approach extends its applicability to a variety of diseases, including ischemic stroke. To investigate the changes in glymphatic function in ischemic stroke, Toh and Siow (87) used the DTI-ALPS index to compare individuals with ischemic stroke and healthy controls. The study reported that the mean DTI-ALPS index ipsilateral to infarct was 1.162 ± 0.126, significantly lower than that for the contralateral side (1.335 ± 0.160). Furthermore, the DTI-ALPS indexes were less impacted in patients who had experienced less severe strokes. Following the initial impairment, DTI-ALPS detected a resurgence after 14 days, indicative of restoration of glymphatic function during the convalescent phase following the acute stage of stroke. They demonstrated that the time interval since the onset of a stroke was a predictor associated with the DTI-ALPS index.

Zhu et al. (55) used the DTI-ALPS index to assess glymphatic function in 18 patients with AIS who achieved recanalization after endovascular treatment. They compared the DTI-ALPS index at 24 h, 72 h, and 7 days post stroke. The results revealed a significantly lower DTI-ALPS index for the infarcted side compared to the contralateral side at both 24 and 72 h. However, this difference disappeared by Day 7. These findings suggest that patients with AIS experience impaired glymphatic function from Day 1 to Day 3 following reperfusion, particularly on Day 3, with partial recovery by Day 7. Furthermore, they investigated the association between the DTI-ALPS index and neurological outcomes. They observed a linear correlation between the DTI-ALPS index and the baseline National Institutes of Health Stroke Scale (NIHSS) scores. Notably, patients with poor outcomes exhibited a significantly lower DTI-ALPS index within 24 h for both the infarcted and contralateral sides compared to those with favorable outcomes. This variation depends on both the functional outcomes and the degree of recanalization. The change in the DTI-ALPS index is influenced by these factors in combination (88).

Furthermore, the DTI-ALPS index has been linked to clinical outcomes in patients with cerebral infarction. Qin et al. (89) investigated the correlation between the DTI-ALPS index and functional impairment in the context of subacute ischemic stroke. They enrolled 26 patients who had experienced subacute ischemic stroke with a single lesion in the left subcortical region and 32 healthy controls, and reported that the left DTI-ALPS index for the ischemic stroke group was significantly lower than that for the healthy controls. In addition, a positive correlation was found between the left DTI-ALPS index and the simple Fugl-Meyer motor function score—a method of assessment specifically designed to evaluate motor function in stroke patients.

These findings suggest that glymphatic system impairment is implicated in the pathophysiology of ischemic stroke. Notably, glymphatic function may change over time in patients who have had an ischemic stroke. Longitudinal research is imperative to elucidate the temporal dynamics of the DTI-ALPS index in patients who have experienced ischemic strokes, as this would facilitate a better understanding of glymphatic function recovery following an initial period of impairment. Chen et al. (90) characterized chronic glymphatic remodeling dynamics using the DTI-ALPS index while exploring its temporal associations with cognitive outcomes. Their results demonstrate that patients who had experienced a stroke had significantly lower DTI-ALPS indices compared to controls at both 3 months and 1 year poststroke. At 3 months, the DTI-ALPS index for the lesioned hemisphere was significantly lower than that for the contralateral hemisphere. However, this difference was no longer observed at 1 year poststroke, indicating that chronic stroke patients exhibit persistent glymphatic dysfunction during the early recovery phase. Lin et al. (91) utilized DTI-ALPS to assess glymphatic system dysfunction in patients with cerebral infarction. Based on their 90-day modified Rankin Scale (mRS) scores, the DTI-ALPS indices were significantly higher for the good prognosis group than for the poor prognosis group. Patients with poor functional outcomes after cerebral infarction exhibit pronounced glymphatic system dysfunction, which correlates with the severity of neurological impairment. These findings suggest that glymphatic impairment may serve not only as a biomarker but also as a putative contributor to adverse stroke outcomes. It is worthwhile to further investigate the correlation between the DTI-ALPS index and the severity of ischemic stroke, as reflected by NIHSS and mRS scores—the NIHSS is a clinical tool used to assess stroke severity at presentation, while the mRS is a widely accepted scale for evaluating long-term functional outcomes following stroke intervention. In addition, it is important to explore how impaired glymphatic function after stroke may manifest in clinical or functional outcomes. Studies that have evaluated the glymphatic system in ischemic stroke using DTI-ALPS are summarized in Table 1.

**Table 1:** Summary of studies evaluating the glymphatic system in ischemic stroke using DTI-ALPS.

Author/year	Participant	Mean age (year) (range)	Time post-stroke	Key findings
Lin et al. (91)	82 poor prognosis group (*n* = 42) good prognosis group (*n* = 40)	51.05 ± 2.81 vs. 50.13 ± 4.72	18.67 ± 2.22 days vs. 19.25 ± 1.74 days	L-ALPS, R-ALPS, and mean-ALPS index were significantly lower in the poor prognosis group compared to good prognosis group
Chen et al. (90)	51 chronic stroke patients	53.25 ± 10.56 (22–77)	3–12 months post-stroke	Significantly lower DTI-ALPS index in stroke patients versus healthy controls at both 3 months and 1 year
Zhu et al. (55)	18 acute ischemic stroke patients	58.3 ± 17.9 (18–86)	Acute ischemic stroke at 24 ± 4 h and 72 ± 12 h	ALPS index of the infarcted side was significantly lower than that of the contralateral side in 24 and 72 h Patients with poor outcomes had a significantly lower ALPS index of both the infarcted and contralateral sides
Chao et al. (98)	96	58.6 ± 12.9	6.7 days	ALPS index was significantly lower in stroke patients than in HCs
Qin et al. (89)	20 subacute patients with single lesion in left subcortical	59.2 ± 12.1	9 (7–40) days	The DTI-ALPS index of the infarcted side in the IS group was significantly lower than the same side in the HC group A higher DTI-ALPS index was associated with a better simple Fugl-Meyer motor function score
Toh and Siow (87)	50	56.7 ± 15.2	17.1 ± 14.8 days	The mean ALPS index of infarct side was significantly lower than that of the contralateral side

mRS, modified Rankin Scale; ALPS, diffusion tensor image analysis along the perivascular space.

## 6 Limitations of DTI-ALPS

Although numerous studies have demonstrated an association between the DTI-ALPS index and various neurological disorders, including stroke, Alzheimer’s disease, Parkinson’s disease, and small vessel diseases (15, 16, 87, 89, 92, 93), the validity of the DTI-ALPS index as a reliable marker of glymphatic brain clearance remains questionable. Manual placement of the region of ROI remains one of the primary limitations of the DTI-ALPS method, as it is susceptible to subjective interpretation and arbitrary decision-making (84). Therefore, standardizing the criteria for ROI placement is essential to minimize variability among observers. Furthermore, the observed association should not be equated with causality because several factors, such as patient motion and blood flow dynamics, may exert a significant influence. In addition, spatial resolution, the specificity of diffusivity metrics, and confounding factors such as white matter pathology and age may influence the interpretation of the DTI-ALPS index. The spatial resolution achievable with normal DTI is far larger than that of tissue structures, including the PVS. The ROIs for calculating the DTI-ALPS index include not only the medullary vessels and PVS but also the surrounding white matter (84, 94). Therefore, it is not possible to evaluate only the diffusivity of the PVS along the medullary vessels. The spatial resolution of standard DTI is insufficient to resolve small structures such as the PVS. Given that the ROIs used to calculate the DTI-ALPS index encompass not only the medullary vessels and PVS but also the surrounding white matter, it is not feasible to selectively assess the diffusivity of the PVS along the course of the medullary vessels. Thus, it is crucial to enhance the resolution and accuracy of DTI imaging protocols to capture the PVS and its associated diffusion dynamics more effectively. Iliff et al. (21) initially described the glymphatic system *in vivo*, and then subsequently employed two-photon laser scanning microscopy to visualize the movement of fluorescent dextrans injected intracisternally from the surface into the cerebral cortex, with very limited penetration into the deep cerebral white matter. This technique offers the benefit of enabling direct visualization of the brain parenchyma but only near the surface of the brain. A subsequent study utilizing intrathecal gadobutrol injection as a CSF tracer corroborated this finding, demonstrating minimal or nonexistent tracer enhancement within regions of deep white matter (95). Multiple functions of the waste elimination system of the brain have been elucidated across distinct anatomical locales. The major problem with the DTI-ALPS method is that it is currently limited to evaluating white matter outside the lateral ventricles, where the medullary arteries and veins intersect with the ventricular wall. The phenomena observable using the DTI-ALPS method are only a fraction of waste clearance in the human brain. It is unlikely using the DTI-ALPS method to characterize the function of the entire glymphatic system. Thus, the relationship between the DTI-ALPS index and glymphatic function must be interpreted with caution. It is advisable to denote a reduced DTI-ALPS index as such, rather than directly attributing it to *glymphatic dysfunction*, in order to avoid inaccurate characterization of the underlying pathophysiology (84). Recently, four-dimensional flow MRI (4D flow MRI) has emerged as a powerful imaging technique that can acquire a large amount of volumetric fluid velocity data with high spatiotemporal resolution. The studies in this domain, which are aimed at characterizing the flow dynamics of CSF, are not only of great significance for clarifying the normal physiological functions of the brain and spinal cord but are also crucial for exploring neurological diseases involving dysfunctions such as impaired clearance of brain metabolic waste (96). Accordingly, integrating diverse methodological approaches is essential for a comprehensive assessment of the glymphatic system, with the anticipated requirement that these methods be applied complementarily in future studies.

## 7 Conclusions and prospectives

The discovery of the glymphatic system enabled a novel mechanistic understanding of pathophysiological processes such as metabolic waste clearance, water transport, and intracranial pressure modulation in CNS diseases. Similarly, the theory of the glymphatic system may provide a new direction for the treatment of neurological diseases. The involvement of glymphatic system dysfunction in ischemic stroke pathogenesis has been established. However, the precise mechanism that underpins this dysfunction and its contribution to ischemic stroke remains unclear. DTI-ALPS can provide potential neuroimaging markers for evaluating impairment of the glymphatic system following an ischemic stroke, with a low DTI-ALPS index implying glymphatic dysfunction. Nonetheless, this noninvasive method has some drawbacks. The other imaging methods currently used to evaluate the glymphatic system also have certain limitations, as they do not capture the entirety of this intricately complex system. Therefore, it is imperative to employ a combination of diverse methodologies to comprehensively evaluate the glymphatic system. Efforts to develop novel imaging tools for human glymphatic system research are commendable, yet must be paired with a prioritized focus on resolving fundamental scientific uncertainties (97).
